# Relationship between triglyceride-glucose index and cognitive function among community-dwelling older adults: a population-based cohort study

**DOI:** 10.3389/fendo.2024.1398235

**Published:** 2024-07-22

**Authors:** Weimin Bai, Shuang An, Hui Jia, Juan Xu, Lijie Qin

**Affiliations:** ^1^ Department of Emergency, Henan Provincial People’s Hospital, People’s Hospital of Zhengzhou University, People’s Hospital of Henan University, Zhengzhou, China; ^2^ Department of Pediatric Rehabilitation, Henan Children’s Hospital Zhengzhou Children’s Hospital, Children’s Hospital Affiliated to Zhengzhou University, Zhengzhou, China; ^3^ Department of Convalescent Four Areas Nine Departments, Navy Qingdao Special Service Recuperation Center, Qingdao, China; ^4^ Department of General Surgery, Affiliated Xiaoshan Hospital, Hangzhou Normal University, Hangzhou, China

**Keywords:** triglyceride glucose, cognitive impairment, elderly, insulin resistance, CHARLS

## Abstract

**Background:**

The global increase in the aging population presents considerable challenges, particularly regarding cognitive impairment, a major concern for public health. This study investigates the association between the triglyceride-glucose (TyG) index, a measure of insulin resistance, and the risk of cognitive impairment in the elderly.

**Methods:**

This prospective cohort study enrolled 2,959 participants aged 65 and above from the 2015 and 2020 waves of the China Health and Retirement Longitudinal Study (CHARLS). The analysis employed a logistic regression model to assess the correlation between the TyG index and cognitive impairment.

**Results:**

The study included 2,959 participants, with a mean age of 71.2 ± 5.4 years, 49.8% of whom were female. The follow-up in 2020 showed a decrease in average cognitive function scores from 8.63 ± 4.61 in 2015 to 6.86 ± 5.45. After adjusting for confounding factors, a significant association was observed between TyG index quartiles and cognitive impairment. Participants in the highest quartile (Q4) of baseline TyG had a higher risk of cognitive impairment compared to those in the lowest quartile (Q1) (odds ratio [OR]: 1.97, 95% confidence intervals [CI]: 1.28–2.62, P<0.001).

**Conclusion:**

The study highlights a significant connection between elevated TyG index levels and cognitive impairment among older adults in China. These findings suggest that targeted interventions to reduce the TyG index could mitigate cognitive impairment and potentially lower the incidence of dementia.

## 1 Introduction

The global aging phenomenon poses unprecedented challenges, notably in aggravating concerns associated with cognitive impairment (1–5). Cognitive impairment encompasses the reception and processing of information, characterized by memory loss, diminished understanding, impaired focus, and difficulties in calculation (6, 7). These cognitive alterations are regarded as the preclinical phase of dementia, possibly affected by factors like fasting glucose levels, physical performance, and other variables, highlighting its significance as a major public health concern (3, 8–10). Therefore, identifying risk factors to prevent cognitive impairment at early stages is essential (3, 6, 11–13).

There is growing evidence supporting the association between insulin resistance (IR) and the risk of cognitive decline (14–17). However, previous studies have primarily relied on the gold standard methods of insulin clamp and intravenous glucose tolerance test for diagnosing IR, which are not commonly performed in clinical settings (18–20).

The triglyceride-glucose (TyG) index, a readily available and cost-effective metric derived from triglyceride (TG) and fasting blood glucose (FBG) levels, has been recognized as a promising surrogate marker for IR (14, 18, 19, 21). Extensive epidemiological research has indicated significant links between the TyG index and various diseases, including cardiovascular diseases, cancers, and diabetes (22, 23). Nonetheless, the validity of the TyG index as an alternative indicator of IR in assessing its relationship with cognitive impairment is still in question. Prior investigations have largely concentrated on the TyG and cognitive function relationship within specific demographics, such as non-diabetic, gender-specific cohorts, or individuals living in rural areas, often focusing on specific cognitive domains (24–27).

This study, grounded in population-based research, seeks to explore the relationship between the TyG index and the occurrence of cognitive impairment in a wider elderly population. Utilizing the most recent cognitive function follow-up data from the 2020 China Health and Retirement Longitudinal Study (CHARLS) database, our work aims to offer an exhaustive and prolonged examination of this association across the entire elderly cohort. The study hypothesis is that elevated TyG index may be associated with cognitive impairment among older adults in China.

## 2 Materials and methods

### 2.1 Study population

**Figure 1:**
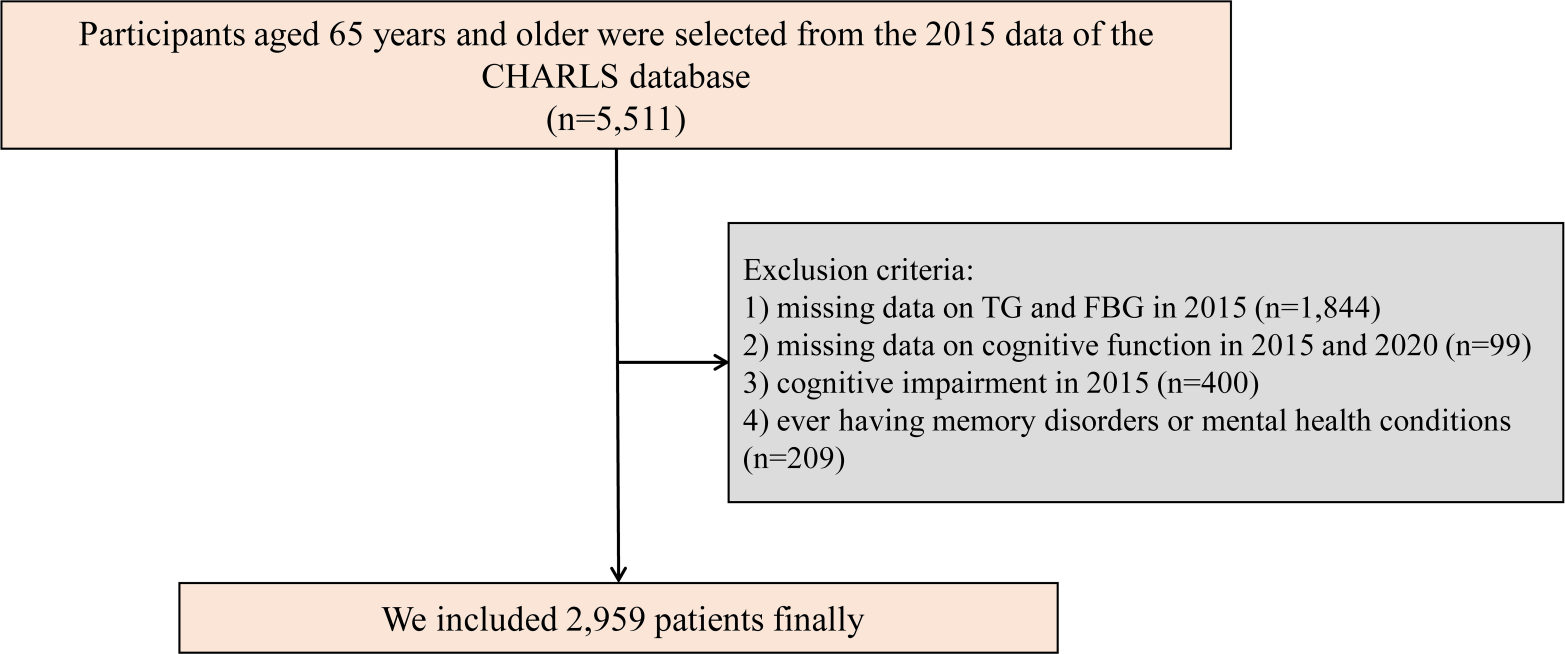
Study flowchart. CHARLS, China Health and Retirement Longitudinal Study; TG, triglyceride; FBG, fasting blood glucose.

The CHARLS is a comprehensive national study comprising five waves aimed at collecting health and social data from Chinese citizens aged 45 years and older (28). The CHARLS project aims to analyze the issue of population aging in China and promote interdisciplinary research on aging. CHARLS is a recurring survey conducted every 2 to 3 years. CHARLS employs a multi-stage probability proportional to size sampling approach, encompassing a sample frame of 450 villages, 150 counties, and 28 provinces. The study involves participation from over 20,000 individuals residing in approximately 10,000 households. Participants undergo face-to-face interviews at home using computer-assisted personal interviewing technology. Survey topics include basic demographic information of respondents and their families, intergenerational transfers within households, health status, medical insurance coverage, employment, income, expenditures, and assets. Additionally, CHARLS includes physical measurements and blood sample collection. Detailed information about CHARLS has been published in previous literature, and the CHARLS dataset is available for download on the CHARLS homepage at http://charls.pku.edu.cn/en. For our study, we used data from the 2015 and 2020 CHARLS surveys, with the former serving as the baseline. Initially, 5,511 participants aged 65 and older were selected from the database (25). To ensure the integrity of the data, we applied specific exclusion criteria: (1) participants with missing data on TG and FBG in 2015 (n = 1,844); (2) participants with missing data on cognitive function in 2015 and 2020 (n = 99); (3) participants with cognitive impairment in 2015 (n = 400); (4) ever having memory disorders or mental health conditions (n = 209). As a result, 2,959 participants satisfied all inclusion criteria and were incorporated into the study (**Figure 1**).

CHARLS received ethical approval from the Ethical Review Committee of Peking University (IRB00001052-11015). All participants provided informed consent by signing consent forms before taking part in the study.

### 2.2 Measurement of cognitive function and TyG index

Assessments of cognitive function were conducted during the follow-up surveys in 2015 and 2020, incorporating tests for episodic memory and mental acuity using a method akin to that employed in the American Health and Retirement Study (28). Participants were first asked to remember a list of ten words immediately after an interviewer read them aloud. Approximately 4 minutes later, they were asked to recall these words once more (delayed recall). The evaluation of episodic memory was based on the average scores from both the immediate and delayed recall tasks, with possible scores ranging from 0 to 10. For assessing mental acuity, participants completed a series of tasks including drawing a specific figure accurately, answering questions regarding the current date, season, and day of the week, and performing a serial subtraction task (subtracting 7 from 100 in five consecutive attempts). Each correct answer earned one point, culminating in a maximum possible score of 11 points (29, 30). The aggregate of these scores represented the participant’s overall cognitive status, with total scores varying between 0 and 21, where higher scores signified superior cognitive function. According to previous research, cognitive impairment was determined as a score 1.0 standard deviation or more below the mean value of cognitive function (31–33).

TG and FBG levels were measured using a standard enzymatic colorimetric method. The TyG index was computed as ln (fasting TG [mg/dL] × FBG [mg/dL]/2) (14, 34). Participants were categorized into four groups (Q1, Q2, Q3, and Q4) based on quartiles of their TyG index.

### 2.3 Covariates

Covariates were selected based on previous research, baseline differences, and clinical significance (26, 33, 35). The potential confounders considered included age, gender, body mass index (BMI), educational level (illiterate, elementary school, middle school, and higher), place of residence (urban and rural), alcohol consumption (more than once a month, less than once a month, never), smoking status (current, former, never), hypertension (yes/no), diabetes (yes/no), overall health status (poor, fair, good, very good, and higher), and cognitive function in 2015.

### 2.4 Statistical analysis

Descriptive statistics were employed to summarize the data, presented as the mean (standard deviation [SD]), median (interquartile range [IQR]), or count and percentage, as appropriate. The chi-square test was used to examine differences among categorical variables. For continuous variables adhering to a normal distribution, one-way analysis of variance (ANOVA) was utilized. When continuous variables did not follow a normal distribution, the Kruskal–Wallis test was applied. These statistical methods aimed to evaluate the disparities and relationships between variables in the study.

Participants were categorized into four groups based on quartiles of the TyG index: Q1 (<8.21), Q2 (≥8.21–8.55), Q3 (≥8.55–8.97), and Q4 (≥8.97). A logistic regression model was used to investigate the independent associations between the TyG index and cognitive impairment, with results presented as adjusted odds ratios (OR) with 95% confidence intervals (CI). Covariates were selected based on previous literature and clinical insight (26, 33, 35). Model 1 was unadjusted; Model 2 adjusted for age and gender; and Model 3 further adjusted for age, gender, BMI, educational level, residence, drinking and smoking status, hypertension, diabetes, overall health, and cognitive function in 2015. We have also utilized Cox regression as a sensitivity analysis to further bolster our research findings. A subgroup analysis also explored the relationship between the TyG index and cognitive impairment across specific subgroups, including gender (male and female), age (65–75 years and ≥75 years), BMI (<24 kg/m^2^ and ≥24 kg/m^2^) (25), presence of chronic diseases (0 and ≥1), and diabetes (yes and no).

The statistical significance was determined using two-tailed tests, with a significance threshold of < 0.05 for the *p* values. All statistical analyses were performed using R software, version 4.2.2, developed by the R Foundation for Statistical Computing, based in Vienna, Austria.

## 3 Results

### 3.1 Baseline characteristics

The current study comprised a cohort of 2,959 participants. The participants’ mean age was 71.2 ± 5.4 years, with 49.8% being female (n = 1,475). They were categorized into four groups according to the quartiles of the TyG index: Q1 (739), Q2 (740), Q3 (740), and Q4 (740). A majority of the participants lived in rural areas (59.5%) and had attained an elementary school level of education (47.8%). The average BMI was recorded at 23.85 ± 7.68 kg/m^2^, and the mean score for depressive symptoms was 8.05 ± 6.50. Furthermore, 32.8% of the participants reported a history of chronic diseases, whereas 56.1% identified as current smokers. Participants in the highest TyG index group displayed a higher BMI, a greater proportion of females, and a more prevalent history of ≥2 chronic diseases, notably diabetes and hypertension. **Table 1** details the baseline characteristics more comprehensively.

**Table 1:** Baseline characteristics according to TyG index level at 2015 wave.

Characteristic	TyG index level					P-value
	Overall	Q1 (<8.21)	Q2 (≥8.21, <8.55)	Q3 (≥8.55, <8.97)	Q4 (≥8.97)	
	N= 2959	N = 739	N = 740	N = 740	N = 740	
Female, n (%)	1475 (49.8)	246 (33.3)	335 (45.3)	419 (56.6)	475 (64.2)	<0.001
Age, years	71.2 (5.4)	71.3 (5.4)	71.4 (5.3)	71.2 (5.4)	70.9 (5.4)	0.327
BMI, kg/m^2^	23.85 (7.68)	22.58 (8.63)	22.52 (3.54)	24.09 (7.54)	26.22 (8.75)	<0.001
Residence, n (%)						0.344
Rural	1441 (59.5)	360 (61.5)	374 (61.1)	352 (58.0)	355 (57.4)	
Urban	981 (40.5)	225 (38.5)	238 (38.9)	255 (42.0)	263 (42.6)	
Educational, n (%)						0.632
Illiterate	1038 (35.1)	251 (34.0)	266 (35.9)	264 (35.7)	257 (34.7)	
Elementary school	1414 (47.8)	367 (49.7)	354 (47.8)	335 (45.3)	358 (48.4)	
Middle school and above	507 (17.1)	121 (16.4)	120 (16.2)	141 (19.1)	125 (16.9)	
Health, n (%)						0.069
Poor	140 (4.8)	29 (4.0)	31 (4.3)	33 (4.5)	47 (6.5)	
Fair	575 (19.9)	133 (18.5)	148 (20.4)	139 (19.1)	155 (21.5)	
Good	1552 (53.6)	384 (53.5)	381 (52.4)	395 (54.3)	392 (54.4)	
Very good and above	627 (21.7)	172 (24.0)	167 (23.0)	161 (22.1)	127 (17.6)	
Marital status, n (%)						0.223
Single	780 (26.4)	189 (25.6)	213 (28.8)	179 (24.2)	199 (26.9)	
Married	2179 (73.6)	550 (74.4)	527 (71.2)	561 (75.8)	541 (73.1)	
History of smoke, n (%)						<0.001
Current	782 (56.1)	248 (57.7)	242 (64.0)	154 (51.5)	138 (48.1)	
Cessation	513 (36.8)	145 (33.7)	125 (33.1)	123 (41.1)	120 (41.8)	
Never	99 (7.1)	37 (8.6)	11 (2.9)	22 (7.4)	29 (10.1)	
History of drink, n (%)						<0.001
More than once a month	742 (25.1)	254 (34.4)	198 (26.8)	164 (22.2)	126 (17.0)	
Less than once a month	214 (7.2)	56 (7.6)	56 (7.6)	45 (6.1)	57 (7.7)	
Never	2002 (67.7)	429 (58.1)	486 (65.7)	530 (71.7)	557 (75.3)	
Depressive score	8.05 (6.50)	7.99 (6.54)	7.98 (6.50)	8.13 (6.60)	8.11 (6.38)	0.960
Chronic diseases, n (%)						0.040
0	1988 (67.2)	523 (70.8)	508 (68.6)	482 (65.1)	475 (64.2)	
1	523 (17.7)	113 (15.3)	125 (16.9)	144 (19.5)	141 (19.1)	
≥2	448 (15.1)	103 (13.9)	107 (14.5)	114 (15.4)	124 (16.7)	
Diabetes, n (%)	318 (10.7)	67 (9.1)	76 (10.3)	76 (10.3)	99 (13.4)	<0.001
Hypertension, n (%)	1223 (41.3)	291 (39.4)	301 (40.7)	307 (41.5)	324 (43.8)	<0.001
Cognitive function in 2015	8.63 (4.61)	8.92 (4.64)	8.67 (4.58)	8.60 (4.59)	8.32 (4.58)	0.031
Cognitive function in 2020	6.86 (5.45)	7.12 (5.47)	7.04 (5.45)	6.87 (5.42)	6.38 (5.32)	0.007

Continuous variables were shown in mean (SD) and categorical variables were shown in percentages.

TyG index, Triglyceride glucose index; BMI, body mass index.

### 3.2 Association between TyG index and cognition function

In the cognitive function assessments conducted in 2015, the average score was 8.63 ± 4.61. A follow-up visit in 2020 indicated a progressive decline in cognitive function scores to 6.86 ± 5.45 (**Table 1**). After adjusting for confounding variables, a significant relationship emerged between the TyG index quartiles and cognitive impairment. Participants in Q4 were found to have a higher risk of cognitive impairment compared to those in Q1 (OR: 1.97, 95% CI: 1.28–2.62, *P* < 0.001). Nevertheless, the associations between the TyG index in Q2 and Q3 quartiles and cognitive impairment did not reach statistical significance in the 2020 follow-up (*P* > 0.05) (**Table 2**).

**Table 2:** The association between TyG index (quartiles) and the risk of cognitive impairment in 2020.

	Event (%)	Model 1 ^a^		Model 2 ^b^		Model 3 ^c^	
		OR (95% CI)	P Value	OR (95% CI)	P Value	OR (95% CI)	P Value
2020 cognitive impairment							
Q1	160 (21.7)	Ref.		Ref.		Ref.	
Q2	168 (22.7)	0.91 (0.72-1.15)	0.436	0.86 (0.69-1.13)	0.330	0.75 (0.47-1.20)	0.231
Q3	176 (23.8)	1.01 (0.72-1.41)	0.671	1.03 (0.73-1.57)	0.609	1.11 (0.70-1.69)	0.634
Q4	188 (25.4)	1.14 (1.02-2.03)	0.014	1.52 (1.19-2.20)	<0.001	1.97 (1.28-2.62)	<0.001
P value for trend			0.007		<0.001		<0.001

TyG index, triglyceride glucose index; OR, odds ratios; CI, confidence intervals.

a unadjusted.

b adjusted for age, gender.

c adjusted for age, gender, body mass index, educational level, residence, drinking status, smoking status, hypertension, diabetes, health status, cognitive function in 2015.

### 3.3 Subgroup and sensitivity analyses

The subgroup analysis demonstrated consistent outcomes across various stratified subgroups, such as gender, age, BMI, chronic diseases, and diabetes, with no significant interaction effects observed (*P*-interaction > 0.05). Participants in the highest quartile of the TyG index (Q4) exhibited an increased risk of cognitive impairment, with the exception of individuals characterized by a lower BMI, absence of chronic diseases, and male gender (**Table 3**). The sensitivity analysis yielded results consistent with the main findings, indicating a significant correlation between TyG index and cognitive impairment (**Additional Table 1**).

**Table 3:** Subgroup and interaction analysis between the TyG index and cognitive impairment in 2020 across various subgroups.

Subgroups	TyG	Event (%)	OR (95% CI)	P Value		TyG	Event (%)	OR (95% CI)	P Value	P-interaction
Gender										0.531
Male	Q1	104 (21.1)	Ref.		Female	Q1	56 (22.8)	Ref.		
	Q2	88 (21.7)	0.73 (0.39-1.28)	0.261		Q2	80 (23.9)	1.34 (0.29-2.95)	0.731	
	Q3	85 (26.5)	0.86 (0.52-1.69)	0.653		Q3	91 (21.7)	1.45 (0.32-3.17)	0.639	
	Q4	70 (26.4)	1.05 (0.59-2.07)	0.118		Q4	118 (25.9)	1.15 (1.05-1.27)	0.003	
Age										0.443
65-75 years	Q1	101 (18.2)	Ref.		≥75 years	Q1	59 (32.0)	Ref.		
	Q2	102 (18.3)	0.81 (0.43-1.49)	0.486		Q2	66 (36.3)	0.62 (0.30-1.24)	0.179	
	Q3	106 (18.9)	1.18 (0.58-2.41)	0.637		Q3	70 (39.1)	1.34 (0.69-2.19)	0.193	
	Q4	116 (20.2)	1.25 (1.01-2.59)	<0.001		Q4	72 (43.6)	1.76 (1.09-3.12)	<0.001	
BMI										0.544
<24 kg/m^2^	Q1	130 (21.4)	Ref.		≥24 kg/m^2^	Q1	30 (24.0)	Ref.		
	Q2	109 (21.5)	0.97 (0.59-1.68)	0.803		Q2	59 (26.5)	1.09 (0.85-1.62)	0.721	
	Q3	93 (24.3)	1.13 (0.90-2.59)	0.523		Q3	83 (23.9)	1.15 (0.78-2.13)	0.565	
	Q4	69 (25.5)	1.46 (0.67-3.51)	0.901		Q4	119 (26.0)	1.51 (1.11-3.52)	<0.001	
Chronic diseases										0.902
0	Q1	130 (21.8)	Ref.		>=1	Q1	30 (20.8)	Ref.		
	Q2	126 (21.7)	0.87 (0.48-1.58)	0.656		Q2	42 (26.3)	0.80 (0.27-2.32)	0.692	
	Q3	122 (22.0)	1.03 (0.66-1.92)	0.271		Q3	54 (29.0)	1.01 (0.32-3.29)	0.753	
	Q4	128 (23.4)	1.20 (0.97-2.05)	0.924		Q4	60 (31.0)	1.23 (1.13-2.78)	<0.001	
Diabetes										0.815
No	Q1	145 (20.5)	Ref.		Yes	Q1	15 (22.4)	Ref.		
	Q2	156 (22.5)	0.93 (0.49-1.78)	0.851		Q2	12 (15.8)	1.02 (0.32-2.01)	0.383	
	Q3	159 (23.7)	1.25 (0.74-2.10)	0.165		Q3	17 (22.4)	1.19 (0.82-2.89)	0.820	
	Q4	156 (27.4)	1.53 (1.17-2.35)	<0.001		Q4	32 (32.3)	1.69 (1.05-3.07)	<0.001	

Adjusted OR in cognitive impairment across quantiles of TyG index by gender, age, BMI, chronic diseases, and diabetes groups. TyG, triglyceride-glucose; BMI, body mass index; OR, odds ratios; CI, confidence intervals.

## 4 Discussion

In this study, we observed a significant association between a higher TyG index and cognitive impairment in older adults (aged 65 years and above). A follow-up visit in 2020 revealed a gradual decline in cognitive function scores compared to the baseline in 2015. Stratified analysis by age, gender, BMI, chronic diseases, and diabetes showed that individuals with a higher TyG index faced a greater risk of cognitive impairment, with the exception of those with lower BMI, those without chronic diseases, and males. These findings suggest that a higher TyG index could be a potential risk factor for cognitive impairment in older adults, particularly in females and in those with higher BMI and chronic diseases.

The aging global population presents an unprecedented challenge, raising significant concerns (1–3). The rapid pace of aging intensifies the challenges associated with cognitive impairment (4, 5). The age-related decline in cognitive function has become a significant public health issue, leading to adverse health outcomes (11, 13). Cognitive impairment often manifests years before the onset of dementia, underscoring the need to explore its mechanisms and to implement preventive measures aimed at risk factors. Previous research has identified several key factors contributing to cognitive decline, including genetic predisposition, cardiovascular disease, and exposure to air pollution (36–40).

IR is characterized by reduced sensitivity and responsiveness to the effects of insulin, acting as a central factor in the emergence of various health issues, such as diabetes, cardiovascular diseases, and cognitive decline (3, 14). Moreover, some evidence suggests that IR is linked to an increased risk of cognitive decline (14–16). The hyperinsulinemic euglycemic clamp, despite its exceptional sensitivity in assessing the body’s response to insulin, is costly and complex, limiting its use in clinical environments. The homeostasis model assessment of insulin resistance (HOMA-IR), based on FPG and insulin measurements, is considered the gold standard for evaluating insulin sensitivity. However, the measurement of insulin levels is not commonly included in routine clinical practice, thereby restricting the use of HOMA-IR in such contexts. The TyG index, a practical measure of IR derived from TG and FBG levels, offers a cost-effective and accessible alternative for IR assessment, gaining widespread adoption in research (14, 18, 19). Epidemiological studies have linked the TyG index to various conditions, including cardiovascular diseases, cancers, and diabetes. Yet, the effectiveness of the TyG index as an indirect marker for IR in exploring its association with cognitive impairment remains to be fully ascertained.

Previous research on the TyG index and cognitive function has predominantly focused on specific demographic groups, such as non-diabetic individuals, gender-specific populations, and rural dwellers, often categorizing cognitive function into distinct domains (24–27). Utilizing data from the National Health and Nutrition Examination Survey (NHANES), Wei et al. found a notable correlation between a high TyG index and reduced cognitive function, as determined by the CERAD test, in non-diabetic elderly individuals in the United States (24). Similarly, a study among elderly residents in rural China linked elevated TyG index values with diminished cognitive performance and brain atrophy (25).

Our population-based study aimed to explore the relationship between the TyG index and cognitive impairment among the elderly population in China. Utilizing data from a continuous cohort, we collected information from consecutive cognitive assessments conducted in 2020. We found a significant association between a higher TyG index and cognitive impairment in older adults during the follow-up visits in 2020, especially pronounced in females, individuals with a higher BMI, and those suffering from chronic conditions. Additionally, our findings showed that cognitive function scores were lower in females than in males. These results align with previous research. Earlier investigations have consistently indicated that females are at a greater risk of cognitive impairments compared to males (3, 41, 42). A study examining a 5-year change in the TyG index and its impact on cognitive function found that females in the second quartile of longitudinal TyG index change exhibited a significant association with reduced cognitive performance as measured by the CERAD test (26). Moreover, it was noted that females have a higher vulnerability to cognitive impairment compared to males (3).

Several factors might elucidate the observed results, necessitating consideration of the underlying mechanisms. Insulin possesses the ability to traverse the blood-brain barrier through specific receptors, affecting both behavioral and metabolic functions (17, 43). IR represents a unique metabolic disorder often characterized by increased insulin levels, which can lead to neurodegeneration and persistent memory impairments due to prolonged exposure of brain neurons to elevated insulin levels (44). Furthermore, IR could diminish cerebral glucose metabolism in particular brain regions, possibly adversely affecting memory function in individuals (45). Some clinical studies have indicated that TG can cross the blood-brain barrier, potentially impairing cognitive function by inducing insulin receptor resistance (46). In our study, most female participants were postmenopausal, a phase associated with reduced estrogen levels. Previous research has identified estrogen as vital for learning and memory, providing neuroprotection and potentially enhancing cognitive function through estrogen therapy (47, 48). This could explain the more pronounced cognitive decline observed in females compared to males in our study. In the BMI-stratified analysis, the link between an elevated TyG index and cognitive impairment was observed solely in overweight and obese individuals, highlighting IR’s traditional association with obesity, which is closely related to brain atrophy (25). Additionally, a study examining the relationship between the TyG index and lower brain volume found this association exclusively in individuals with a BMI ≥24 kg/m^2^ (25).

The issue of cognitive impairment is becoming increasingly evident as the population ages. Serving as an early indicator of dementia, cognitive impairment is associated with a notably adverse prognosis, affecting individual quality of life and placing burdens on families and society. The early prevention of cognitive impairment is thus critical and urgent. Our research suggests that the TyG index could serve as an alternative marker of IR for predicting cognitive impairment in individuals over 65 years old in China, with a significant relationship observed between high TyG index and cognitive impairment. Targeted interventions could help in mitigating cognitive impairment, potentially decreasing the incidence of dementia. Additionally, further exploration into identifying more predictive risk factors for cognitive impairment is necessary to achieve the objective of early prevention.

This study has several limitations. First, it is important to recognize that the study was observational. Despite efforts to adjust for known confounders, the potential impact of unmeasured confounders on the outcomes cannot be disregarded. Therefore, the applicability of our findings may be somewhat restricted. Moreover, we evaluate cognitive function using episodic memory and mental acuity rather than clinical diagnosis. Comprehensive assessment across multiple dimensions is crucial for understanding overall cognitive function, highlighting limitations when evaluating solely based on episodic memory and mental acuity. However, these neuropsychological tests are widely regarded as reliable screening tools for measuring cognitive function and are extensively used in clinical practice (49). Finally, due to data availability limitations, our analysis was confined to a 5-year follow-up period from 2015 to 2020. Future studies should consider employing repeated-measures designs over longer durations and examining more potential pathways. Subsequent research is warranted to refine these findings further.

## 5 Conclusion

The results of this study reveal a notable association between elevated TyG index levels and the occurrence of cognitive impairment among the elderly Chinese demographic. The initiation of targeted intervention strategies may effectively mitigate cognitive impairment, potentially decreasing the prevalence of dementia.

## Data availability statement

The datasets presented in this study can be found in online repositories. The names of the repository/repositories and accession number(s) can be found below: http://charls.pku.edu.cn/pages/data/111/zhcn.html.

## Ethics statement

The studies involving humans were approved by the Ethical Review Committee of Peking University (IRB00001052-11015). The studies were conducted in accordance with the local legislation and institutional requirements. Written informed consent for participation was not required from the participants or the participants’ legal guardians/next of kin in accordance with the national legislation and institutional requirements.

## Author contributions

WMB: Data curation, Formal analysis, Investigation, Methodology, Validation, Writing – original draft. SA: Data curation, Investigation, Project administration, Validation, Writing – original draft. HJ: Data curation, Formal analysis, Investigation, Project administration, Software, Writing – original draft. JX: Conceptualization, Formal analysis, Methodology, Resources, Supervision, Writing – review & editing. LJQ: Data curation, Formal analysis, Investigation, Methodology, Resources, Supervision, Writing – review & editing.
