# Supplementary material for: Relationship between triglyceride-glucose index and cognitive function among community-dwelling older adults: a population-based cohort study
Source: Front Endocrinol (Lausanne). 2024 Jul 22;15:1398235. doi: 10.3389/fendo.2024.1398235 (PMC11298491; doi:10.3389/fendo.2024.1398235)
Supplement: Supplementary file 1 [file Table_1.docx]

**Additional Table 1 The association between TyG index (quartiles) and the risk of cognitive impairment in 2020**

|  | Event (%) | Model 1 ^a^ | | Model 2 ^b^ | | Model 3 ^c^ | |
| --- | --- | --- | --- | --- | --- | --- | --- |
|  |  | HR (95% CI) | P Value | HR (95% CI) | P Value | HR (95% CI) | P Value |
| **2020 cognitive impairment** | |  | |  | | | |
| Q1 | 160 (21.7) | Ref. |  | Ref. |  | Ref. |  |
| Q2 | 168 (22.7) | 1.01 (0.82-1.30) | 0.126 | 1.17 (0.93-1.28) | 0.210 | 1.25 (0.98-1.41) | 0.412 |
| Q3 | 176 (23.8) | 1.09 (0.62-1.47) | 0.750 | 1.23 (0.72-1.53) | 0.719 | 1.31 (0.79-1.72) | 0.647 |
| Q4 | 188 (25.4) | 1.21 (1.05-1.93) | <0.001 | 1.64 (1.11-2.32) | <0.001 | 2.07 (1.54-2.92) | <0.001 |
| P value for trend |  |  | <0.001 |  | <0.001 |  | <0.001 |

TyG index = triglyceride glucose index; HR = hazard ratios; CI = confidence intervals.

^a^ unadjusted

^b^ adjusted for age, gender

^c^ adjusted for age, gender, body mass index, educational level, residence, drinking status, smoking status, hypertension, diabetes, health status, cognitive function in 2015
