# Supplementary material for: Relationship between triglyceride-glucose index and cognitive function among community-dwelling older adults: a population-based cohort study
Source: Front Endocrinol (Lausanne). 2024 Jul 22;15:1398235. doi: 10.3389/fendo.2024.1398235 (PMC11298491; doi:10.3389/fendo.2024.1398235)
Supplement: Supplementary file 2 [file Table_2.docx]

**Additional Table 2 Baseline characteristics of participants aged 65 years and older: included vs. excluded**

| **Characteristic** | **Included Participants** | **Excluded Participants** | **P-value** |
| --- | --- | --- | --- |
|  | **N= 2959** | **N = 2552** |  |
| Female, n (%) | 1475 (49.8) | 1210 (47.4) | 0.076 |
| Age, years | 71.2 (5.4) | 71.7 (5.7) | 0.337 |
| BMI, kg/m^2^ | 23.85 (7.68) | 22.98 (7.82) | 0.575 |
| **Residence, n (%)** |  |  | 0.282 |
| Rural | 1441 (59.5) | 1480 (58.0) |  |
| Urban | 981 (40.5) | 1072 (42.0) |  |
| **Educational, n (%)** |  |  | 0.733 |
| Illiterate | 1038 (35.1) | 880 (34.5) |  |
| Elementary school | 1414 (47.8) | 1215 (47.6) |  |
| Middle school and above | 507 (17.1) | 457 (17.9) |  |
| **Health, n (%)** |  |  | 0.010 |
| Poor | 140 (4.8) | 105 (4.1) |  |
| Fair | 575 (19.9) | 454 (17.8) |  |
| Good | 1552 (53.6) | 1483 (58.1) |  |
| Very good and above | 627 (21.7) | 510 (20.0) |  |
| **Marital status, n (%)** |  |  | 0.068 |
| Single | 780 (26.4) | 618 (24.2) |  |
| Married | 2179 (73.6) | 1934 (75.8) |  |
| **History of smoke, n (%)** |  |  | 0.114 |
| Current | 782 (56.1) | 680 (59.5) |  |
| Cessation | 513 (36.8) | 375 (32.8) |  |
| Never | 99 (7.1) | 87 (7.7) |  |
| **History of drink, n (%)** |  |  | 0.001 |
| More than once a month | 742 (25.1) | 689 (27.0) |  |
| Less than once a month | 214 (7.2) | 253 (9.9) |  |
| Never | 2002 (67.7) | 1610 (63.1) |  |
| Depressive score | 8.05 (6.50) | 7.98 (5.95) | 0.560 |
| **Chronic diseases, n (%)** |  |  | 0.543 |
| 0 | 1988 (67.2) | 1679 (65.8) |  |
| 1 | 523 (17.7) | 467 (18.3) |  |
| ≥2 | 448 (15.1) | 406 (15.9) |  |
| Diabetes, n (%) | 318 (10.7) | 242 (9.5) | 0.133 |
| Hypertension, n (%) | 1223 (41.3) | 1018 (39.9) | 0.290 |
| Cognitive function in 2015 | 8.63 (4.61) | 7.90 (4.02) | 0.217 |
| Cognitive function in 2020 | 6.86 (5.45) | 5.79 (4.34) | 0.079 |

Continuous variables were shown in mean (SD) and categorical variables were shown in percentages.

**Abbreviation:** BMI = body mass index.
